# Message in a Bottle: Dialog between Intestine and Skin Modulated by Probiotics

**DOI:** 10.3390/ijms18061067

**Published:** 2017-06-09

**Authors:** Adrián D. Friedrich, Mariela L. Paz, Juliana Leoni, Daniel H. González Maglio

**Affiliations:** 1 Universidad de Buenos Aires, Facultad de Farmacia y Bioquímica, Cátedra de Inmunología, 1053 Buenos Aires, Argentina; mlpaz@ffyb.uba.ar; 2 CONICET-Universidad de Buenos Aires, Instituto de Estudios de la Inmunidad Humoral (IDEHU), LIBAP, 1053 Buenos Aires, Argentina; jleoni@ffyb.uba.ar

**Keywords:** probiotics, immunomodulation, ultraviolet radiation, skin immune system, photoimmunology

## Abstract

At the beginning, probiotics were used exclusively for gastrointestinal conditions. However, over the years, evidence has shown that probiotics exert systemic effects. In this review article, we will summarize recent reports that postulate probiotic treatment as an efficient one against skin pathologies, such as cancer, allergy, photoaging and skin infections. The focus will be restricted to oral probiotics that could potentially counteract the ultraviolet irradiation-induced skin alterations. Moreover, the possible underlying mechanisms by which probiotics can impact on the gut and exert their skin effects will be reviewed. Furthermore, how the local and systemic immune system is involved in the intestine-cutaneous crosstalk will be analyzed. In conclusion, this article will be divided into three core ideas: (a) probiotics regulate gut homeostasis; (b) gut and skin homeostasis are connected; (c) probiotics are a potentially effective treatment against skin conditions.

## 1. Introduction

Three billion microorganisms live within the human body, and they constitute what we know as the microbiota. The largest and most diverse niche is the bowel, which has very special conditions. Many types of microorganisms, each one in a particular amount, can live there without generating an inflammatory microenvironment. In fact, during the last decade, it became clear that the microbiota and the host establish a symbiosis, which has been crucial for our evolution as a species. Thus, our whole physiology depends on the relationship we build with the microbiota. Particularly, the intestinal microbiota is essential for all mammal nutrition, synthesizing vitamins that the host cannot produce by himself/herself, digesting complex carbohydrates and taking part in amino acid homeostasis (especially important in ruminants and coprophage mammals) [1].

In 1989, Strachan published an epidemiological study where 17,000 individuals were included and followed for 23 years. He found an inverse relationship between hay fever incidence and the number of siblings of the analyzed individuals. Strachan hypothesized that “These observations could, however, be explained if allergic diseases were prevented by infection in early childhood, transmitted by unhygienic contact with older siblings, or acquired prenatally from a mother infected by contact with her older children. Later infection or reinfection by younger siblings might confer additional protection against hay fever” [2]. This work is considered as the foundation of the “hygiene hypothesis”, which in turn incorporated autoimmune diseases [3] into allergic disorders as the pathologies modulated by early microbial challenges.

Later in time, many researchers proposed highly industrialized food consumption, anti-microbiotic abuse, as well as commensal parasites’ elimination as factors responsible for altering the intestinal microbiota. They observed that several autoimmune diseases and allergies developed more frequently if those factors were present in the patients. Experiments performed using germ-free animals (animals born and raised in sterile conditions and therefore microbiota-free) have proven that the microbiota is crucial for the development, maturation and normal function of the immune system [4].

The establishment of a beneficial intestinal microbiota would not just contribute to intestinal homeostasis, but also might have desirable systemic effects by reducing the incidence of certain pathologies. Moreover, it has become clear that there is a relationship between dysbiosis, inflammation and cancer. Mice genetically manipulated to develop inflammatory bowel disease (IBD) can pass on ulcerative colitis (UC) [5], as well as colorectal cancer [6] when co-housed with wild-type animals, by horizontal and vertical transmission of pathogenic microbes. At the same time, the microbiota is implicated in the immune response against non-intestinal tumors. In a simple but elegant experiment published in 2015, Sivan and col proved that genetically-identical animals purchased in different laboratories were colonized by different bacteria and responded differently to the B6 melanoma cell line implanted in their skin. In fact, the administration of fecal suspensions equalized the tumor growth between animals [7]. These results suggest a connection between the intestinal microbiota and immune response against skin tumors.

To summarize, the integrity of intestinal microbiota seems to be one of the key points for the normal development and function of the immune system, while an unbalanced microbiota would predispose to pathology. In the present review article, we will present the current knowledge of the worldwide most-studied strategy to induce a sustained beneficial microbiota: probiotics. Moreover, we will present bibliographic evidence to prove that probiotics could serve as a therapeutic approach for skin conditions

## 2. Microbiota and Probiotics

The Russian zoologist Ilia Metchnikoff, 1908 Nobel Prize awardee, published in 1907 the book “The Prolongation Of Life: Optimistic Studies”. There, he proposed that large amounts of fermented food consumption could be related to the longevity observed in elderly people of the Caucasus [8]. He was one of the first researchers that presented lactic bacteria, and fermented food produced by them, as beneficial nutrients and as useful therapeutic tools for many pathologies.

On the other hand, the World Health Organization defined probiotics as live microorganisms that when administered in adequate amounts, confer a benefit to health [9]. Along these few past years, it has been shown, both in experimental animal models and human individuals, that the beneficial effects depend strongly on the species of the probiotic tested. Just to give some examples, *Lactobacillus reuteri* and *Lactobacillus casei*, but not *Lactobacillus plantarum*, prime monocyte-derived dendritic cells (DC) in a tolerogenic fashion [10]. Besides, in a dextran-induced ulcerative colitis (UC) model, *Lactobacillus rhamnosus*, but not *Bifidobacterium breve*, rapidly and effectively improved the dextran-induced bloody diarrhea during the resolution phase [11]. At last, in a double blind clinical study, *L. rhamnosus* HN001, but not *B. animalis*, reduced eczema incidence when compared to children administered with placebo [12].

Once ingested, viable probiotics have to reach the intestinal lumen, colonize and, from that location, produce their effects. Despite that probiotics may not alter the microbiota composition in healthy adults [13,14], they appear to re-establish the microbiota more rapidly after antimicrobial therapy [15]. Besides, in elderly individuals, certain probiotics affect the microbiota metabolism, like flagellar motility, chemotaxis and the adhesion of intestinal bacteria [16]. The question of whether those effects do impact on human health is still open.

## 3. Probiotics and Gut-Associated Lymphoid Tissue

The gut-associated lymphoid tissue (GALT) is the most complex of all local immune compartments. It is composed of an epithelial barrier constituted mainly by enterocytes, intraepithelial T lymphocytes, M cells and Paneth cells, a specialized connective tissue called lamina propria, constituted by highly diverse cell lineages (like macrophages, DC, plasmocytes, T-cells), known as the effector compartment of the GALT, and Peyer’s patches (PP) and mesenteric lymph nodes (MLN) known as the induction compartment of the GALT.

There are many articles that explore the effects of probiotics on the GALT; some of them are reviewed by Corthésy [17]. Briefly, the author summarizes that the effects observed are due to whole bacteria, as well as soluble factors that impact on enterocyte pattern recognition receptors (PRRs), like Toll-like receptors. Upon recognition, enterocytes secrete TGF-β and IL-8, which in turn modulate the immune response. Despite the fact that the WHO dictates that microorganisms must be viable to be considered as probiotics, there is evidence that shows that inactivated or heat-killed bacteria, as well as isolated purified molecules, can exert effects on the GALT [18,19,20,21,22,23]. In fact, it has been suggested to name these soluble probiotic-derived molecules as “probiotaceuticals” [24].

Besides the enterocytes, DC are crucial for probiotic-host interactions by extending interdigitating dendrites to intestinal lumen and sensing microbial antigens. Afterwards, they mature, migrate and prime T-cells in PP and MLN, inducing different activation profiles. These activated T-cells promote, ultimately, B-cell switching and IgA secretion to the intestinal lumen. Once more, the effects will depend on the microorganisms analyzed and the experimental model used. Young mice administered orally with *L. acidophilus* show an upregulation of co-stimulatory molecules in MLN DC. Moreover, when injected in non-treated animals, these DC show an increase in their migration rate to MLN and colon. Finally, when mice were challenged with *Citrobacter rodentium*, an intestinal pathogen, these DC significantly reduced the infection susceptibility [25].

In conclusion, probiotics generate immunomodulation in many tissues and cells of the GALT. Broadly speaking, probiotics induce a protective state against intestinal infections by modulating enterocytes, DC, T- and B-cells.

## 4. Skin Microbiota and the Skin Immune System

The skin is the largest tissue in the human body and, similar to the intestine, is colonized by millions of microorganisms, around one million bacteria per square centimeter. On the one side, the skin immune system (SIS) is similar to the GALT, as it is a local and organized part of the immune system and is composed of an epithelium, an effector and an induction compartment, as well. On the other side, there are differences in each tissue of the SIS. The epithelium is composed mainly by keratinocytes, but it also contains melanocytes, Langerhans cells (LC) and intraepithelial T-cells; the effector compartment, the dermis, as the lamina propria, is constituted by many different cell types; and the induction of the response happens in the skin draining lymph nodes (SLN).

As in the GALT, the SIS homeostasis depends strongly on the skin microbiota. In fact, the skin is exposed to a great amount of physical, chemical and biological agents that could alter the balance between the microbiota and the SIS. Skin dysbiosis is associated not only with opportunistic microbial infection, but also with chronic skin conditions, especially atopic dermatitis (AD) [26] and psoriasis [27]. Recently, a study published in *Nature* demonstrated that dysbiosis is also present in a non-inflammatory skin pathology, vitiligo [28]. Thus, it is becoming clear that alternative therapeutical strategies to preserve the normal skin microbiota are potentially interesting approaches to treat diverse skin conditions. It is important to remark that there is still no evidence that could clearly establish if the dysbiosis is a cause or a consequence of skin pathologies here mentioned.

Apart from preventing microbial infection, the SIS is involved in inducing an anti-inflammatory or tolerogenic state in normal conditions that allows the tissue to coexist with the skin microbiota peacefully. The hyperactivity of different cell populations in the SIS, like keratinocytes, DC and specific T-cells, is present in different skin conditions, like psoriasis [29], atopic eczema (AE) [30] and contact dermatitis (CD) [31,32].

The first clinical evidence that has shown connections between skin pathology and intestinal microbiota alterations emerged more than a decade ago. Small intestine bacterial overgrowth (SIBO) is more frequent in rosacea patients than in healthy individuals. Moreover, the treatment and eradication of SIBO in rosacea patients resolve rosacea manifestations in 26 of 28 of the individuals analyzed [33]. On the other hand, inflammatory bowel diseases, like UC and Crohn’s disease, often present skin manifestations. A review article published in 2012 summarizes this issue and presents some of the mechanisms involved [34].

## 5. Skin Immune System and Probiotics

On the basis of the above, probiotics can result in a potentially interesting therapeutic strategy for skin clinical conditions. For that purpose, probiotics may be used topically or orally. Despite the fact that there are some articles that show beneficial effects of probiotics on human keratinocytes in vitro [35,36,37] and on mouse skin [38], there are only a few clinical trials showing effectiveness of topical treatment. One of them proved that *L. plantarum* HY7714 applied during 12 weeks on the skin of volunteers aged 41 to 59 years, with dry skin and wrinkles, improved photoaging parameters [39]. In addition, a preliminary study shows that topical application of *L. plantarum* in burn wounds reduces skin infection risk. This constitutes a novel bacteriotherapy for this condition [40].

On the other hand, orally-administered probiotics have been studied profusely for different skin conditions. Regarding AD, there are more clinical trials performed in children than in adult patients, probably because this pathology is more prevalent in this group [41]. Meneghin and col gathered together clinical trials where different probiotics were used to prevent or treat AD in children: 13 out of 17 studies showed effectiveness in the prevention of AD, while 15 out of 20 showed effectiveness in the treatment of this condition [42]. The differences observed in each clinical trial may be explained by the diversity of the probiotic organisms used (including different species of the *Lactobacillus* and *Bifidobacterium* genus and combinations of them), the clinical conditions and the parameters evaluated. Kukkonen and col performed a double-blind, randomized clinical trial administering placebo or capsules containing probiotics to 1223 pregnant women, carrying children with high risk for allergy, for two to four weeks. These capsules include a combination of *L. rhamnosus* GG, *L. rhamnosus* LC705, *B. breve* Bb99 and *Propionibacterium freudenreichii* ssp. *shermanii* JS. The newborns received the same capsules, but with the prebiotics galacto-oligosaccharides or placebo, during six months. The study revealed that there were no differences in allergic skin conditions, while the incidence of AE in two-year-old children was reduced [43]. Similar results were obtained with *L. rhamnosus* alone [12], but opposed to the latter, *L. reuteri* has been shown to reduce the incidence of IgE-dependent eczema in children [44]. When tested in adult AD patients, *B. animalis* subsp. *lactis* LKM512 [45], heat-killed *L. acidophilus* L-92 [46] and *L. salivarius* LS01 [47] were each shown to have improved AD symptoms. 

## 6. Probiotics and Cancer

In relation to cancer therapy, the clinical use of probiotics is controversial. To date, there are no clinical trials where probiotics were used as adjuvant therapy for cancer, even though many original articles have been written about probiotics’ effects in experimental models, both in vitro and in vivo. Most of the work on humans is epidemiologic. There, the incidence of colorectal cancer, in the first place, and bladder and mammary cancer, in the second, is correlated with nutritional habits, including the consumption of fermented food [48]. *The Lancet* published an epidemiological study performed in Denmark and Finland, which showed a reduced incidence of colon cancer in a rural population, a fact that could be explained by higher milk product consumption, including fermented food. In fact, the low-incidence population had higher numbers of *Lactobacillus* in feces [49]. Moreover, an epidemiological study performed in the USA showed a correlation between higher yogurt consumption and a reduced colon cancer incidence [50]. At last, another U.S. epidemiological study suggests that cultured milk consumption in adults could be protective for colon cancer development [51].

There are, also, reports of probiotics’ efficacy in the treatment of extra-intestinal cancer in animal models. Balb/C mice orally administered with *L. acidophilus*, before and after mammary carcinoma cell line injection, showed a higher survival rate compared to the placebo group [52]. Furthermore, *L. helveticus* yogurt was shown to reduce and abolish the tumor development in a mammary cancer mouse model, after seven, but not two, days of probiotic treatment [53].

However, what is the state of knowledge about oral probiotic therapy in skin cancer? Sadly, there are few original articles that explore this field. In a brilliant recent paper, Silvan and col demonstrated the effectiveness of an oral *Bifidobacterium* cocktail for melanoma treatment, in a mouse model. They have shown that the oral probiotic is as efficient as PDL-1-specific monoclonal antibody (mAb), the clinically-approved checkpoint blockade therapy. In fact, the co-administration of both the anti-PDL1 mAb and the *Bifidobacterium* cocktail almost abolished tumor growth. This is the first report of a mixed treatment of oral probiotics and regular chemotherapy, in an effort to improve the efficacy of the latter [7]. Furthermore, in our laboratory, we have demonstrated that oral administration of purified lipoteichoic acid (LTA) from *L. rhamnosus* GG retards and reduces UV-induced skin squamous cell carcinomas, in a mouse model of chronic UV irradiation [54].

## 7. Ultraviolet Radiation, Probiotics and Cancer

The biological effects of ultraviolet radiation (UVr), in particular the UVB fraction, have been deeply studied during the last century. Briefly, it is widely known that the most important direct effects on exposed cells are the DNA pyrimidine dimer formation and the isomerization of urocanic acid. Moreover, reactive oxygen and nitrogen species are important byproducts induced after UV irradiation. The most important distant or systemic effect is the photo-immunosuppression described for the first time by the group of Dr. Margaret Kripke [55,56]. Thanks to their work in the late 1970s, knowledge has been built about UVr inducing an immunosuppressive state, which is both local (at the skin level), as well as systemic. This leads to an impaired immune surveillance, which, in turn, can favor cancer development [57]. In addition, there is clinical evidence that shows that UVr is strongly related to photoaging [58,59,60]. In the last few years, oral probiotic treatment has been proposed as an interesting alternative approach for photoprotection, which may be useful in reducing photoaging, UV-induced immunosuppression and cancer development.

Pequet-Navarro and col performed a clinical study with 54 healthy volunteers, who received *L. johnsonii* or placebo during six weeks prior to sun-simulated-UV irradiation. They reported that Langerhans cells from probiotic-treated individuals showed an increased repopulation of the skin, as well as a functional recovery four days after irradiation, compared to the placebo group [61]. In another study, *L. johnsonii* was mixed with carotenoids and subsequently administered to healthy individuals, before they were exposed to “non-extreme UV with high UVA level”. As a result, inflammatory dermal cells (CD45+) were reduced, Langerhans cells’ reduction was avoided and dermal dendrocytes (XIIIa+ cells) were increased in treated individuals, compared to the placebo-administered group [62]. However, it is not possible to distinguish if the effect observed was due to the probiotics, to the carotenoids or to the combination of both. The same probiotic strain, *L. johnsonii*, was employed by Guéniche and col in an animal model of UV-induced immunosuppression. They demonstrated that treatment with the probiotic prior to the irradiation challenge results in a restoration of the cellular immune response.

Less studied are the UVr effects on skin microbiota. Each microorganism in the skin has particular susceptibilities to UVr, which depend also on the time and intensity of the radiation. This could alter the microbiota composition in exposed individuals [63].

## 8. Possible Mechanisms Involved in the Skin Effects of Oral Probiotic Treatment

Given the intestinal barrier impermeability to microbial cells and microbial-derived molecules, and therefore the improbable passage of those antigens to the blood, it is feasible to hypothesize the following mechanisms involved in transmitting probiotic signals to the host: (a) probiotics or probiotic-derived molecules could modulate the systemic immune system by activating DC [7,61], stimulating NK cells [62,63] and inducing different T-cell differentiation profiles/subsets [64,65]; or (b) they could modulate the intestine-brain-skin axis.

We will focus on those original articles that explore the skin effects of oral-administered probiotics, setting aside the enormous bibliography that proves in vitro probiotic effects.

### 8.1. Mechanisms Involved in the Systemic Immunomodulation by Oral Probiotics

Scientists have tried to understand probiotic-host interactions at the skin level by using either contact hypersensitivity (CHS) or delayed-type hypersensitivity (DTH). *L. casei* DN-114 001 has been shown to reduce both dinitrofluorobenzene (DNFB)-induced CHS, as well as Ovoalbumin-induced DTH in healthy animals. The mechanism described is related to a reduction in cytotoxic T-cells’ chemotaxis to challenged skin, an increase in regulatory T-cells’ (Tregs) differentiation and an augmented recruitment of these regulatory cells to the challenged skin. In addition, Tregs purified from spleen and draining lymph nodes of probiotic-treated and DNFB-sensitized animals showed a three-fold increase in IL-10 secretion after in vitro polyclonal stimulation. By an adoptive transfer experiment, CD8+ T-cells isolated from probiotic-treated animals were shown to produce a decreased CHS reaction in CD3^−/−^ recipients compared to CD8+ T-cells from placebo-treated group. On the contrary, a co-transfer experiment showed no differences in CHS reaction between Tregs from the probiotic-treated group and from the control animals in CD3^−/−^ recipients. Altogether, these results demonstrate that this particular probiotic induces an impaired CD8+ T-cell function, but did not alter Tregs’ effector function [66]. In an AD mouse model, where the pathology was already established, an oral cocktail containing *L. casei*, *L. plantarum*, *L. rhamnosus* and *B. lactis* managed to reduce IgE levels, as well as IL-4 and IL-5, while increasing IL-12p40 and IFN-γ. Treated mice also showed reduced mastocyte infiltration and degranulation [67]. This evidence shows that probiotics are able not only to modulate T-cell subsets’ activation during normal immune responses, but they also may modulate soluble mediators in established pathological conditions.

In relation to the mechanisms that could explain the reduction in UVr-induced skin damage by oral probiotics, as mentioned above, it has been shown that *L. johnsonii* treatment could re-establish LC turn over after irradiation in humans [61] and abolish UV-induced immunosuppression in mice [68]. Both effects could be explained, at least in part, by a reduction in IL-10 blood level after UVr in probiotic-treated animals. On the other side, *L. plantarum* administration could decrease MMP-13 expression and activity in hairless mice, thus reducing UVr-induced photoaging [69].

Regarding the DC role in oral probiotic skin effects, Sivan and col demonstrated that melanoma implanted animals administered with a *Bifidobacterium* cocktail had more activated DC (increased MHCII expression) infiltrating the tumor microenvironment than saline solution-treated ones. In addition, those DC were able to activate cytotoxic T-cells more efficiently and induced higher IFN-γ levels in vitro. The authors concluded that signals produced by *Bifidobacterium*, but not the bacteria itself, could induce DC activation in the steady state. In turn, this could lead to an improved specific cytotoxic T-cell function. They suggest that this effect could be occurring in a non-antigen-specific fashion [7]. Similarly, in our laboratory, we have proven that LTA from *L. rhamnosus* GG administered to chronically UV-irradiated animals increases CD4+ and CD8+ T-cells, as well as it increases IFN-γ secretion in SLN [54].

Besides the articles here reviewed, many original papers have been written, which describe probiotics’ effects in the GALT. However, to date, there is no work centered on oral probiotic mechanisms that could explain, on one side, the effects seen in the GALT and, on the other side, the modulation of the SIS. Here, some questions that are still open are presented, with regards to the mechanisms mentioned above:

(1) Are the mechanisms working in a non-antigen-specific fashion and therefore due to the innate immune system, or, on the contrary, are there antigens both present in damaged skin and in probiotic-conditioned intestine that are being presented in the GALT and therefore responding in an antigen-specific fashion?
(2) The effector cells, as DC and T-cells, appear to be involved in the effects observed in the skin after probiotic oral treatment. Are these cells coming from the GALT, or are there just cytokines that are secreted in the GALT, reaching the skin through blood and activating those cells in the SIS?
(3) Are there bone marrow-derived DC or inflammatory DC taking part in these effects?
(4) How is it explained that the same probiotic bacteria can, on one side, reduce inflammatory levels during an established AD and, on the other side, improve a pro-inflammatory antitumoral response?

### 8.2. Mechanisms Involving the Intestine-Brain-Skin Axis

The intestine-brain-skin axis hypothesis was first proposed 80 years ago by the dermatologists John H. Stokes and Donald M. Pillsbury [70] and has recently become of interest once again. Briefly, the hypothesis is centered on the capacity of emotional states (e.g., depression and anxiety) to alter normal intestinal microbiota, to increase intestinal permeability and to contribute to systemic inflammation. They were also among the first ones to propose the use of probiotic *L. acidophilus* cultures for human health. Even if the present review article focus is the link between oral probiotics and skin, the brain could be considered an organ taking an important part in this crosstalk [71].

It has been postulated that substance P (SP) could be involved in the skin-intestine crosstalk. This could be explained by: (a) skin conditions like AD are characterized by a great amount of neuroinflammation with high levels of SP in the skin [72]; (b) oral antibiotic therapy induces SP release in the enteric nervous system, while *L. paracasei* oral treatment reduces this release [73]. Although there are well-known similarities and connections between the skin and intestine nervous systems and both of them together with the brain have a common embryologic origin, more evidence is needed in order to fully understand the relation between oral probiotics and the intestine-brain axis.

## 9. Conclusions

Fermented food has been consumed by worldwide cultures since at least the year 6000 B.C. Scientific evidence around the beneficial effects of its consumption is not a novelty. However, the mechanisms involved in the probiotic-host interactions are still poorly understood. After years of deep research, it has become clear that the effects observed depend profusely on the particular microorganisms evaluated. In the future, it may be possible to choose the precise microorganism or combination for each pathology. Last, but not least, probiotics may be potentially explored as an adjuvant treatment to improve the effectiveness of different therapeutic approaches.
